# The Dacini fruit fly fauna of Sulawesi fits Lydekker’s line but also supports Wallacea as a biogeographic region (Diptera, Tephritidae)

**DOI:** 10.3897/zookeys.973.55327

**Published:** 2020-10-05

**Authors:** Camiel Doorenweerd, Arni Ekayanti, Daniel Rubinoff

**Affiliations:** 1 University of Hawaii, College of Tropical Agriculture and Human Resources, Department of Plant and Environmental Protection Sciences, Entomology section, 3050 Maile Way, Honolulu, Hawaii, 96822-2231, USA University of Hawaii Honolulu United States of America; 2 Niogret Ecology Consulting LLC, Wotu, Luwu Timor, Sulawesi Selatan 92971, Indonesia Niogret Ecology Consulting LLC Wotu Indonesia

**Keywords:** *
Bactrocera
*, biogeography, *
Dacus
*, pest, taxonomy, *
Zeugodacus
*, zoogeographic

## Abstract

Although there is scientific consensus on most of the major biogeographic regions in the world, the demarcation of the area connecting Southeast Asia with Australia and Oceania remains debated. Two candidate boundaries potentially explain faunistic diversity patterns in the regions: Lydekker’s and Wallace’s lines. The islands in between both ‘lines’ are jointly termed Wallacea, with Sulawesi as the largest landmass. We surveyed Dacini fruit flies (Tephritidae: Dacinae) in Sulawesi between 2016 and 2019 using traps baited with male lures, resulting in 4,517 collected flies. We identified all specimens to species level, which adds 15 new species records to the island, bringing the total number of Dacini species in Sulawesi to 83. The biogeographic affinity of species in the updated checklist reveals a strong connection with former ‘Sunda’ (41% of species); validating Lydekker’s line, but also a high level of endemism (47% of species), confirming the uniqueness of Wallacea as a biogeographic region. We further describe a new species, Bactrocera (Bactrocera) niogreta Doorenweerd, **sp. nov.** and discuss the taxonomy of several interesting species.

## Introduction

**Figure 1.**
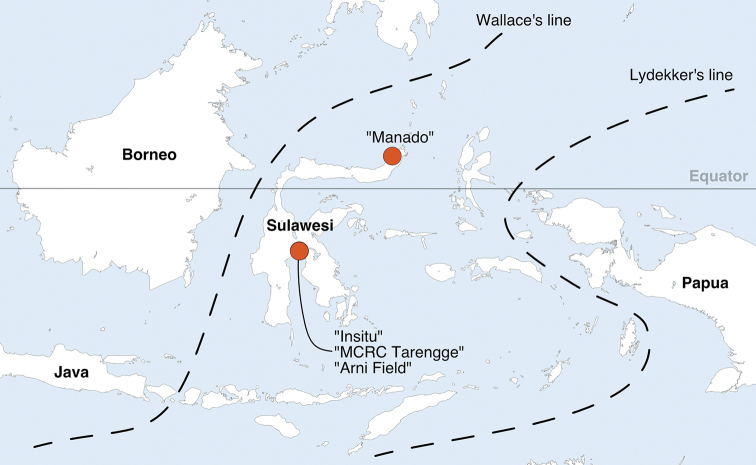
Map of Sulawesi and neighboring areas showing the four sampling localities with orange spots; the three localities in South Sulawesi were in close proximity to each other. Two typical biogeographical boundaries are indicated with dotted lines: Wallace’s line and Lydekker’s line. Land masses west of Wallace’s line were connected during ice ages as Sunda, east of Lydekker’s line land masses were connected as Sahul. Islands in between the two biogeographical boundaries were never connected by land and are jointly known as Wallacea.

Biogeographic boundaries were initially established to indicate stark and sudden differences between faunas of neighboring areas, as noted by early explorers like Alfred Russel Wallace, and proved fundamental to the understanding of tectonic plate movement (Wallace 1876; Lydekker 1896; Mayr 1944; Simpson 1977; Whitmore 1982). Today, they incorporate phylogenetic considerations and define biogeographical regions that can be of broad practical use including for regional identification keys, for understanding dispersal patterns, and designation of biogeographic hotspots for critical conservation considerations (Kreft and Jetz 2010; van Welzen et al. 2011; Holt et al. 2013). The area that connects Southeast Asia with Australia – also termed the Malay Archipelago or Malesia – probably contains the largest number of named biogeographic boundaries anywhere on the planet (Simpson 1977). The two designations that have held up best following extensive studies of both fauna and flora are known as Wallace’s line and Lydekker’s line, with the area in between often referred to as Wallacea (Fig. 1).

Wallace’s line runs south of the Philippines, east of Borneo and continues south between Bali and Lombok (van Welzen et al. 2011). The boundary was hypothesized in works by Wallace (1860), and not long after the term “Wallace’s line” was coined by Huxley (1868). Islands and landmasses west of Wallace’s line are jointly termed the Sunda Shelf and were intermittently connected by land during the Pleistocene ice ages, up to as recently as 21,000 years ago when the sea level was as much as 120 m below current levels (for a review see van Welzen et al. 2011). Lydekker’s line, on the other hand, suggests an alternative separation that would potentially explain the broader faunistic diversity patterns in the regions better. It was proposed by Lydekker (1896), but its significance became more recognized in later studies (Simpson 1977). This boundary runs west of Papua and north of Australia. Papua and Australia are located on the Sahul Shelf and were connected by land during roughly the same periods where Sundaland existed. There has been much debate on which line more accurately indicates the changes in biodiversity composition in the Indomalayan region (Simpson 1977; van Welzen et al. 2011). In some studies, the area between Wallace’s and Lydekker’s lines has been termed Wallacea and interpreted as a separate biogeographic region altogether, as it generally has high levels of endemism. Sulawesi is the largest island in Wallacea, where it is joined with the Moluccas and the lesser Sunda Islands. The area has known substantial geological turmoil: Sulawesi was separated into three parts until the late Miocene (~ 10 Ma). It was cut into the West Sulawesi ophiolite and North Sulawesi ophiolite that have a closer geographic affinity to Eurasia, and the East Sulawesi ophiolite, which was geographically closer to Australia (Hall 1998; Spakman and Hall 2010). The exact timing of the joining of these fragments is still uncertain, partly because it is unclear which areas were submerged in the past 10 Ma (Hall 2009). In any case, the geographic history of the islands has undoubtedly played a large role in the evolution of the fauna of the Sulawesi.

Dacini fruit flies (Tephritidae: Dacinae) are a tribe of 938 described exclusively Old World species (e.g., Doorenweerd et al. 2018). They are mostly known for their potential to damage fruit crop production, as the majority of species are frugivorous and the larvae will feed on many fleshy fruits also used for human consumption (Vargas et al. 2015; Ekesi et al. 2016). The taxonomic and phylogenetic insights in the group have only recently begun to stabilize (Schutze et al. 2015, 2017; Dupuis et al. 2018; San Jose et al. 2018a), and there are likely many species yet undescribed. The first recorded Dacini fruit flies from Sulawesi were four species collected by Wallace in the mid 19^th^ century (Hardy 1982). After those initial collections, the Dacini fauna of the island went unstudied for over a century until Hardy recorded 34 species during a sabbatical leave in 1975 (Hardy 1982). Many of those species were new to science and are endemic to Sulawesi. More recent studies have added further records (Drew and Romig 2013; Drew and Hancock 1994), and this current study adds another 15, which brings the total of Dacini fruit fly species known from Sulawesi to 83. We here provide a checklist of all species, describe Bactrocera (Bactrocera) niogreta sp. nov. as new to science and discuss new species forms, and assess the faunistic affinity of Sulawesi Dacini with neighboring biogeographic areas. In the last comprehensive overview, Hardy wrote: “The *Dacus* [ed: now Dacini] of Sulawesi fit more closely with the fauna of the Australian Region than with that of the Oriental”. The Australian region in this sense included the Moluccas and Papua, thus agreeing with Wallace’s line. We re-evaluate this statement based on the updated species list.

## Materials and methods

### Sampling

We collected Dacini flies using handmade bottle traps. A 3 cm-diameter hole was cut 15 cm from the base of a 500 ml plastic water bottle. Male attractant lures: methyl eugenol 10 g cones (Scentry Biologicals Inc., Billings MT, USA), cue lure 2 g cones (Scentry Biologicals Inc., Billings MT, USA), and zingerone (Sigma-Aldrich, St. Louis MO, USA) were individually suspended by a string inside the bottle, 5 cm from the top. A 100 ml water solution of Fisherbrand Sparkleen detergent (Fisher Scientific, Pittsburgh, PA, USA) poured at the bottom of each bottle trap was used as a killing agent. The traps were then hung from a cacao tree (*Theobroma cacao* L.) branch at 1.5 m high. Traps were checked every 2–5 days, and trapped flies were transferred to 95% ethanol. Trapping was mainly conducted in Wotu, Kabupaten Luwu Timur, South Sulawesi at sites named “Insitu” [WGS84 N 2.5587 E 120.7935], “MCRC Tarengge” [WGS84 N 2.5547 E 120.8047] and “Arni field” [WGS84 N 2.5587 E 120.7935] (Fig. 1).

Planting at Insitu was composed primarily of cacao clones PBC123 and BR25 that were planted 3.5 m apart within a row, with 3.5 m spacing between rows, irregularly shaded by a diversity of fruit trees. This site was the most diversified among the trapping sites, including more than 100 banana (*Musa* sp.), four large durian tree (*Durio* sp.), eight rambutan (*Nephelium lappaceum* L.), six coconut trees (*Cocos nucifera* L.), as well as some ginger (*Alpinia* sp.), Luffa (*Luffa acutangula* L.), papaya (*Carica papaya* L.), chili peppers (*Capsicum* sp.) and corns. The cacao trees were not regularly pruned but were treated with an unknown pesticide, and were not artificially irrigated. This farm was surrounded by neighboring cacao farms with a similar diversified composition. In addition, some jackfruit (*Artocarpus heterophyllus* Lam.), mango (*Mangifera indica* L.), guava (*Psidium guavaja* L.), rose apple (*Syzygium* sp.), as well as breadfruit (*Artocarpus altilis* (Parkinson) Fosberg) were present around the neighboring farms. The site at MCRC Tarengge represents a 1 ha of cacao trees of clone M01 with a 1.5 × 3 m density, without any other fruit trees within the block. However, several langsat trees (*Lansium parasiticum* (Osbeck) Sahni & Bennet), banana and a couple of durian trees were present in the neighborhood farms 100 m away from the trapping sites, as well as 20 papaya, 10 rambutan trees (*Nephelium lappaceum* L.) within 200 m, and several mango trees, jackfruit, guava, and rose apple trees within 400 m radius from the trapping site. No pesticide was applied during our field collection, but both surrounding blocks were regularly treated with pesticides. The site ‘Arni field’ was also mainly composed of cacao trees at lower density (3 × 3 m). Various fruit trees disseminated around the farm, including some banana, rambutans, jackfruit, mangos, guava, and Ambarella (*Spondias dulcis* L.), with rows of corn (*Zea mays* L.) and several durian trees within 50 m, as well as jambu putik (*Syzygium* sp.), rose apple, and breadfruit within 300 m.

In total, the trapping effort at Arni field was approximately four months, five months at MCRC Tarengge, and six and half months at Insitu, spread over different periods during 2016–2019 (Suppl. material 1: Table S1). The site “Manado” [WGS84 1.3973N, 124.6488E], near the city of Manado in North Sulawesi, had three trapping days. At all sites combined, we collected 4,517 Dacini flies and identified all specimens to species level, initially based on external morphology. In cases where the morphology was inconclusive, we used DNA sequences of Cytochrome C Oxidase I and/or Elongation Factor 1-alpha for a total evidence identification approach. In 2016, some additional collecting was done with torula yeast dissolved in water, which attracts females. A full overview of all traps and localities can be found in Suppl. material 1: Table S1. All voucher material is stored at the University of Hawaii Insect Museum (**UHIM**). Photographs of adult specimens were taken using a Zeiss Discovery.V8 stereomicroscope with an attached Sony alpha-6300 camera. Photographs from multiple focal plains were combined into a single stacked image using Affinity Photo 1.7.3 and optimized for publication. Plates with multiple images were assembled in Affinity Designer 1.7.3. Wings of selected specimens were removed and mounted in euparal on glass-slides and photographed in a similar manner as the adults.

### DNA extraction, PCR, sequencing, and analyses

Methods for DNA extraction, PCR primers and conditions, and Sanger sequencing follow those of San Jose et al. (2018a). For the present study, we sequenced a Cytochrome C Oxidase I (COI) 809 base pair 3P’ fragment and an Elongation Factor 1-alpha (EF1-alpha) 762 bp gene fragment for *Bactrocera niogreta*. We compared the sequences to our (partially unpublished) sequence database and here release sequences of the most closely related species to establish the diagnostic discrimination of COI and EF1-a sequences. We also sequenced COI and EF1-a for several specimens of *Bactrocera melastomatos* Drew & Hancock, 1994 and *Dacus longicornis* (Wiedemann, 1830), to confirm if the different morphological forms were mirrored in mitochondrial and/or nuclear genetic variation. Finally, we sequenced EF1-alpha for the two specimens of *B. carambolae* Drew & Hancock, 1994, which is diagnostic at five positions, to confirm its identity (see also Leblanc et al. 2019). All specimen collecting details and DNA sequences are available through BOLD dataset DOI: http://dx.doi.org/10.5883/DS-DACSU, and GenBank accessions: MT456325–MT456363 [COI] and MT456286–MT456324 [EF1-alpha]). We performed maximum likelihood analyses for each subset of sequences using IQTree 1.6.10 (Nguyen et al. 2015). We allowed IQTree to determine the substitution model via its integrated modeltest and ran maximum likelihood analyses with 5,000 ultrafast bootstraps and 5,000 Sh-aLRT bootstraps. We consider branches with support values > 95 % for ultrafast bootstraps and > 80 % for Sh-aLRT bootstraps as well supported. Resulting trees were optimized for publication using FigTree 1.4.3 and Affinity Designer 1.7.3.

## Results

We list 83 species of Dacini for Sulawesi (Table 1): 51 species of *Bactrocera*, 7 *Dacus*, and 25 *Zeugodacus*. We collected 29 species during our surveys, of which 15 are new island records. The biogeographic affinity of most species is with the Sunda region; 34 of the species in the checklist can also currently be found in areas formerly connected under Sunda. This is in stark contrast with affinities related to Sahul; only one species is currently also found there, and an additional single species is found across Sunda, Wallacea and Sahul (*B. umbrosa* (Fabricius, 1805); see also Krosch et al. 2018). All 47 other species are endemic to Wallacea, and 39 of those are known from Sulawesi only, indicating the high levels of endemicity of the region, even for these volant insects.

**Table 1.** Checklist of Dacini in Sulawesi.

Species	Sulawesi record	Male lure	Insitu	MCRC Tarengge	Arni Field	Manado	Biogeographic affinity
*B. abbreviata* (Hardy, 1974)	This study	ZN	x		x		Sunda
*B. affinibancroftii* Drew & Romig, 2013	Drew and Romig 2013	ME					Sulawesi endemic
*B. affinidorsalis* (Hardy, 1982)	Hardy 1982	CL					Sunda
*B. albistrigata* de Meijere, 1911	Drew and Romig 2013	CL	x	x	x	x	Sunda
*B. beckerae* (Hardy, 1982)	Hardy 1982	CL					Sulawesi endemic
*B. bifasciata* (Hardy, 1982)	Hardy 1982	CL					Wallacea
*B. bitungiae* Drew & Romig, 2013	Drew and Romig 2013	CL					Sulawesi endemic
*B. carambolae* Drew & Hancock, 1994	This study	ME	x				Sunda
*B. careofascia* Drew & Romig, 2013	Drew and Romig 2013	CL					Sulawesi endemic
*B. commensurata* Drew & Romig, 2013	This study	ME	x	x	x		Sunda
*B. curvosterna* Drew & Romig, 2013	Drew and Romig 2013	CL					Sulawesi endemic
*B. dispar* (Hardy, 1982)	Hardy 1982	–					Sulawesi endemic
*B. dorsalis* (Hendel, 1912)	Drew and Romig 2013	ME	x	x	x	x	Sunda
*B. elongata* Drew & Romig, 2013	Drew and Romig 2013	CL					Sulawesi endemic
*B. flavipennis* (Hardy, 1982)	Hardy 1982	CL					Sulawesi endemic
*B. flavosterna* Drew & Romig, 2013	Drew and Romig 2013	CL					Sulawesi endemic
*B. floresiae* Drew & Hancock, 1994	Drew and Romig 2013	ME					Sunda
*B. fuscitibia* Drew & Hancock, 1994	Drew and Romig 2013	CL/ZN*					Sunda
*B. fuscolobata* Drew & Romig, 2013	Drew and Romig 2013	CL	x				Sulawesi endemic
*B. fuscoptera* Drew & Romig, 2013	Drew and Romig 2013	ME					Sulawesi endemic
*B. hantanae* Tsuruta & White, 2001	This study	CL	x				Sunda
*B. infulata* Drew & Hancock, 1994	Drew and Hancock 1994	ME					Sulawesi endemic
*B. involuta* (Hardy, 1982)	Hardy 1982	CL					Sulawesi endemic
*B. latifrons* (Hendel, 1915)	Drew and Romig 2013	–					Sunda
*B. limbifera* (Bezzi, 1919)	Drew and Romig 2013	CL	x	x		x	Sunda
*B. linduensis* Drew & Romig, 2013	Drew and Romig 2013	CL	x				Wallacea
*B. megaspilus* (Hardy, 1982)	Hardy 1982	CL			x		Sulawesi endemic
*B. melastomatos* Drew & Hancock, 1994	This study	CL	x	x	x		Sunda
*B. moluccensis* (Perkins, 1939)	Drew and Romig 2013	CL/ZN					Sunda
*B. nanoarcuata* Drew & Romig, 2013	Drew and Romig 2013	CL					Sulawesi endemic
*B. nationigrotibialis* Drew & Romig, 2013	Drew and Romig 2013	ME					Sulawesi endemic
*B. neoritsemai* Drew & Romig, 2013	Drew and Romig 2013	CL					Sulawesi endemic
*B. niogreta* Doorenweerd sp. nov.	This study	ZN	x				Sulawesi endemic
*B. ochroma* Drew & Romig, 2013	Drew and Romig 2013	ME					Sunda
*B. pendleburyi* (Perkins, 1938)	This study	ZN		x	x		Sunda
*B. penebeckerae* Drew & Romig, 2013	Drew and Romig 2013	–					Wallacea
*B. penecostalis* Drew & Romig, 2013	Drew and Romig 2013	CL					Sulawesi endemic
*B. perkinsi* (Drew & Hancock, 1981)	This study	CL	x				Sahul
*B. pernigra* Ito, 1983	This study	CL					Sunda
*B. propinqua* (Hardy & Adachi, 1954)	This study	CL	x				Sunda
*B. pseudobeckerae* Drew & Romig, 2013	Drew and Romig 2013	CL					Sulawesi endemic
*B. ritsemai* (Weyenbergh, 1869)	Drew and Romig 2013	CL					Sunda
*B. splendida* (Perkins, 1938)	This study	ZN*	x				Sunda
*B. sulawesiae* Drew & Hancock, 1994	Drew and Hancock 1994	ME					Sulawesi endemic
*B. suliae* Drew & Romig, 2013	Drew and Romig 2013	ME					Wallacea
*B. syzygii* White & Tsuruta, 2001	This study	ZN	x	x	x		Sunda
*B. terminifer* (Walker, 1860)	Drew 1989	–					Sulawesi endemic
*B. trifasciata* (Hardy, 1982)	Hardy 1982	CL					Sulawesi endemic
*B. umbrosa* (Fabricius, 1805)	Drew and Romig 2013	ME	x	x	x	x	Sunda; Sahul
*B. usitata* Drew & Hancock, 1994	This study	CL	x				Sunda
*B. wuzhishana* Li & Wang, 2006	Drew and Romig 2013	ME					Sunda
*D. donggaliae* Drew & Romig, 2013	Drew and Romig 2013	CL					Sulawesi endemic
*D. longicornis* (Wiedemann, 1830)	Walker 1860; Drew 1989	CL	x				Sunda
*D. melanopectus* Drew & Romig, 2013	Drew and Romig 2013	ME					Sulawesi endemic
*D. nanggalae* Drew & Hancock, 1998	Drew and Hancock 1998	CL					Sulawesi endemic
*D. ortholomatus* Hardy, 1982	Hardy 1982	–					Sulawesi endemic
*D. pedunculatus* (Bezzi, 1919)	This study	ZN*	x	x			Sunda
*D. pullus* (Hardy, 1982)	Hardy 1982	ZN*	x				Sulawesi endemic
*Z. abnormis* (Hardy, 1982)	Hardy 1982	CL					Sunda
*Z. angustifinis* (Hardy, 1982)	Hardy 1982	CL	x				Sulawesi endemic
*Z. apicalis* (de Meijere, 1911)	Hardy 1982	CL	x				Sunda
*Z. bogorensis* (Hardy, 1983)	Hardy 1982	CL					Sunda
*Z. buruensis* (White, 1999)	Hardy 1982	CL					Wallacea
*Z. connexus* (Hardy, 1982)	Hardy 1982	–					Sulawesi endemic
*Z. cucurbitae* (Coquillett, 1899)	Drew and Romig 2013	CL	x	x			Sunda
*Z. dubiosus* (Hardy, 1982)	Hardy 1982	CL					Sulawesi endemic
*Z. emittens* (Walker, 1860)	Walker 1860; Drew 1989	CL					Wallacea
*Z. eurylomatus* (Hardy, 1982)	Hardy 1982	–					Sulawesi endemic
*Z. exornatus* (Hering, 1941)	Drew and Romig 2013	CL	x				Sunda
*Z. flavipilosus* (Hardy, 1982)	Drew and Romig 2013	CL					Sulawesi endemic
*Z. fulvipes* (Perkins, 1938)	Hancock and Drew 2017	CL					Sunda
*Z. hancocki* (Drew & Romig, 2013)	Drew and Romig 2013	CL					Sulawesi endemic
*Z. heinrichi* (Hering, 1941)	Hering 1941	CL/ZN					Sunda
*Z. melanopsis* (Hardy, 1982)	Hardy 1982	CL					Sulawesi endemic
*Z. neoflavipilosus* (Drew & Romig, 2013)	Drew and Romig 2013	CL					Sulawesi endemic
*Z. neolipsanus* (Drew & Romig, 2013)	Drew and Romig 2013	CL					Wallacea
*Z. persignatus* (Hering, 1941)	Drew and Romig 2013	CL	x				Wallacea
*Z. proprescutellatus* (Zhang Che & Gao, 2011)	This study	CL	x				Sunda
*Z. synnephes* (Hendel, 1913)	Drew and Romig 2013	CL					Sunda
*Z. tebeduiae* (Drew & Romig, 2013)	Drew and Romig 2013	CL					Sunda
*Z. transversus* (Hardy, 1982)	Hardy 1982	CL	x				Sulawesi endemic
*Z. ujungpandangiae* (Drew & Romig, 2013)	Drew and Romig 2013	CL					Sulawesi endemic
*Z. vargus* (Hardy, 1982)	Hardy 1982	CL					Sulawesi endemic

*: new lure record. Male lure abbreviations: ME = methyl eugenol, CL = cue lure, ZN = zingerone.

We report four new male lure records of species attracted to zingerone: *Bactrocera splendida* (Perkins, 1938), *B. fuscitibia* Drew & Hancock, 1994 (attracted to both cue lure and zingerone), *Dacus pedunculatus* (Bezzi, 1919), and *D. pullus* (Hardy, 1982). Although the three localities “Insitu”, “MCRC Tarengge” and “Arni Field” are geographically within two kilometers of each other, Insitu had a distinctly higher diversity with 28 species, whereas we only collected ten species at MCRC Tarengge, and nine at the Arni Field, with similar collecting efforts. We collected only four species at the “Manado” site, but this is likely due to less trapping days, and possibly because this was a less forested site just 50 m from the coastline. The major, widely distributed, pest species *B. albistrigata* (de Meijere, 1911) and *B. dorsalis* (Hendel, 1912) were present at all sites and made up 70.6 % of all specimens collected (Suppl. material 1: Table S1).

Below, we describe two new species, provide more information on the first records of *B. carambolae* for Sulawesi, and discuss the presence of *B. melastomatos*. We also describe the second specimen ever collected of *Dacus pullus*, and provide morphological and molecular evidence for two species forms of *Dacus longicornis*.

### Bactrocera (Bactrocera) niogreta

Taxon classificationAnimaliaDipteraTephritidae

Doorenweerd sp. nov.

C1E835A6-1167-5D95-B857-E578E47D20DB

http://zoobank.org/AEC5FE4F-A4F4-4C48-AEB6-27A1A74B6F58

Figures 2–7

#### Holotype.

Male. Labelled: “Indonesia: Sulawesi: South Sulawesi: Insitu. WGS84 -2.5464 120.7921 16–23.i.2019 Zingerone trap. Leg. Jerome Niogret. DNA sample ms09121”. Deposited at the University of Hawaii Insect Museum (UHIM).

#### Differential diagnosis.

Bactrocera (Bactrocera) niogreta sp. nov. is most similar to B. (Tetradacus) brachycera (Bezzi, 1916), which is known from India, Bhutan, and China (Drew and Romig 2013). Both species have an incomplete black ‘T’ marking on the abdomen, and a costal band that follows vein R_4+5_ and expands distally to reach vein M. *Bactrocera niogreta* can be distinguished by the connection of the yellow presutural marking with the notopleuron, which resembles a yellow curly bracket ‘{‘ in dorsal view. *Bactrocera niogreta* further has smaller facial spots, not filling the basal ½ of the socket, and in the male genitalia it has a deep emargination of sternum V, which is shallow in *B. brachycera*. *Bactrocera niogreta* may in Sulawesi be most easily be confused with *B. megaspilus*, but the latter has a more angular expansion of the costal band, no medial black markings on the abdomen, no presutural yellow markings and all fulvous legs.

#### Molecular diagnostics.

The COI sequence of *Bactrocera niogreta* is, in our database, most similar to *Bactrocera fuscitibia*, which can morphologically easily be distinguished by not having a clearly expanded costal band. The EF1-alpha sequences are most similar to *B. enochra* (Drew, 1972), which is morphologically different in not having a wide costal band, and has a wide red band medially across the scutum and three longitudinal black bands along the abdomen. Both COI and EF1-alpha are diagnostic to identify *B. niogreta* (See BOLD Dataset DOI: http://doi.org/10.5883/DS-DACSU).

#### Description of adult.

**Figures 2–7.**
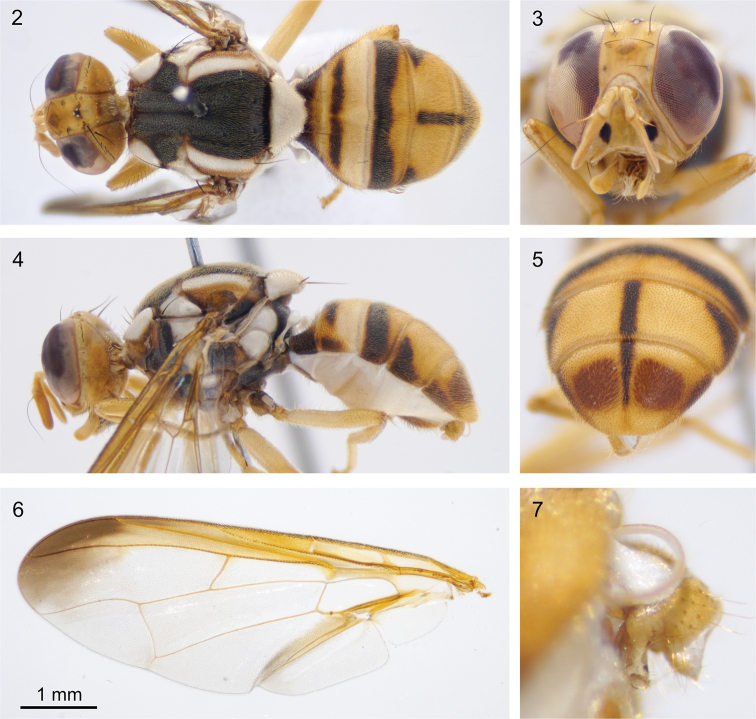
Bactrocera (Bactrocera) niogreta sp. nov. Holotype, ms09121 **2** dorsal view **3** frontal view of the face **4** lateral view **5** posterior view of the abdomen showing the ceromatae **6** dissected wing **7** lateral close-up of the genitalia.

***Head*** (Fig. 3). All parts uniformly fulvous to yellow, ocellar triangle slightly darker. Face fulvous with rectangular spot in each antennal furrow. Antennae uniformly fulvous. ***Thorax*** (Figs 2, 4). Scutum and pleural areas black with narrow red-brown areas lateral of the yellow postsutural lateral vittae. Yellow markings: postpronotal lobes; notopleura; postsutular lateral vittae broad and parallel sided, reaching intra-alar seta; presutural marking to the lateral vittae that connects to the notopleura and in dorsal view resembles a curly bracket ‘{‘; broad mesopleural stripe, almost reaching posterior level of postpronotal lobe, continuing onto katepisternum as a broad transverse spot, anterior margin slightly convex; katatergite; anatergite. Medial vitta absent. Scutellum yellow except for narrow black basal band. Setae: two scutellar; one prescutellar; one intraalar; one posterior supraalar; one anterior supraalar; one mesopleural; two notopleural; four scapular; all setae well developed and red-brown. ***Abdomen*** (Figs 2, 5). Oval to diamond shaped; terga free; pecten present on tergum III; posterior lobe of surstylus short (Fig. 7); abdominal sternum V with a deep concavity on posterior margin that reaches the center of the sternum. Tergum I fulvous with apical margin narrowly yellow. Tergum II yellow with anteromedial dark marking. Tergum III mostly dark, with a narrow concave posterior fulvous band. Terga IV and V with a medial longitudinal dark marking. Tergum IV with triangular anterolateral dark markings, tergum V with narrow anterolateral dark markings. Ceromatae (shining spots) contrasting red-brown. ***Legs*** (Fig. 4). All leg segments fulvous to yellow; tibiae fulvous with apical black spur on mid tibiae; tarsi fulvous to yellow. ***Wings*** (Fig. 6). Length 6.1 mm, basal costal and costal cells fuscous, increasingly darker distally; microtrichia in outer corner of cell costal only; remainder of wings with a pale fulvous tint except fuscous subcostal (anal) cell; broad fuscous costal band that reaches vein R_4+5_, gradually darker distally until dark brown and expands to reach vein M; a broad fuscous anal streak ending at apex of A_1_ + CuA_2_; dense aggregation of microtrichia around A_1_ + CuA_2_; supernumerary lobe not pronounced.

#### Male lure.

Zingerone.

#### Host plant.

Unknown.

#### Etymology.

The species name is an adjective that refers to instigator of the 2016–2019 Dacini surveys in Sulawesi: Jerome Niogret.

#### Comments.

the morphology of *B. niogreta* overall most closely resembles B. (Tetradacus) brachycera, the combination of a short posterior lobe of the surstylus in the male genitalia and a deep concavity on sternum V support placement in subgenus Bactrocera. In the Drew and Romig (2016) key to the Southeast Asian fruit flies, *B. niogreta* characters lead to the Indian species Bactrocera (Bactrocera) andamanensis, couplet 90 on page 140. The key can there be adapted to include that *B. niogreta* differs from *B. andamanensis* in having an all-black scutum, broad postsutural yellow lateral vittae, and dark lateral markings on abdominal segment IV, with no dark markings on the legs.

##### Dacus longicornis form icariiformis

**Figures 8–13.**
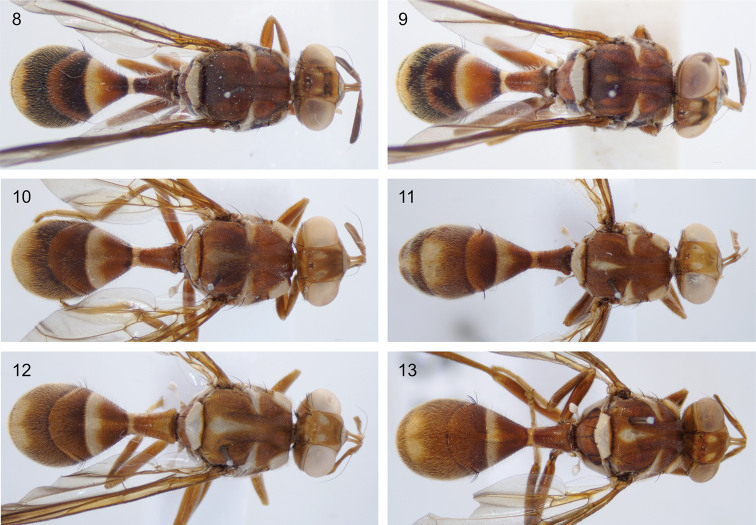
Two forms of *Dacus longicornis***8***D. longicornis* collected in Bangladesh, Pabna district, 30-ix-3-x-2013 Leg. M. A. Hossain **9***D. longicornis* collected in Bangladesh, Maulvi Bazar Rainforest resort, Leg. L. Leblanc & M. A. Hossain **10** specimen ms08424, collected in Sulawesi, with a faint medial postsutural yellow vitta **11** specimen ms08432, collected in Sulawesi **12** specimen ms08428, collected in Sulawesi **13** specimen ms08421, collected in Sulawesi.

**Figures 14, 15.**
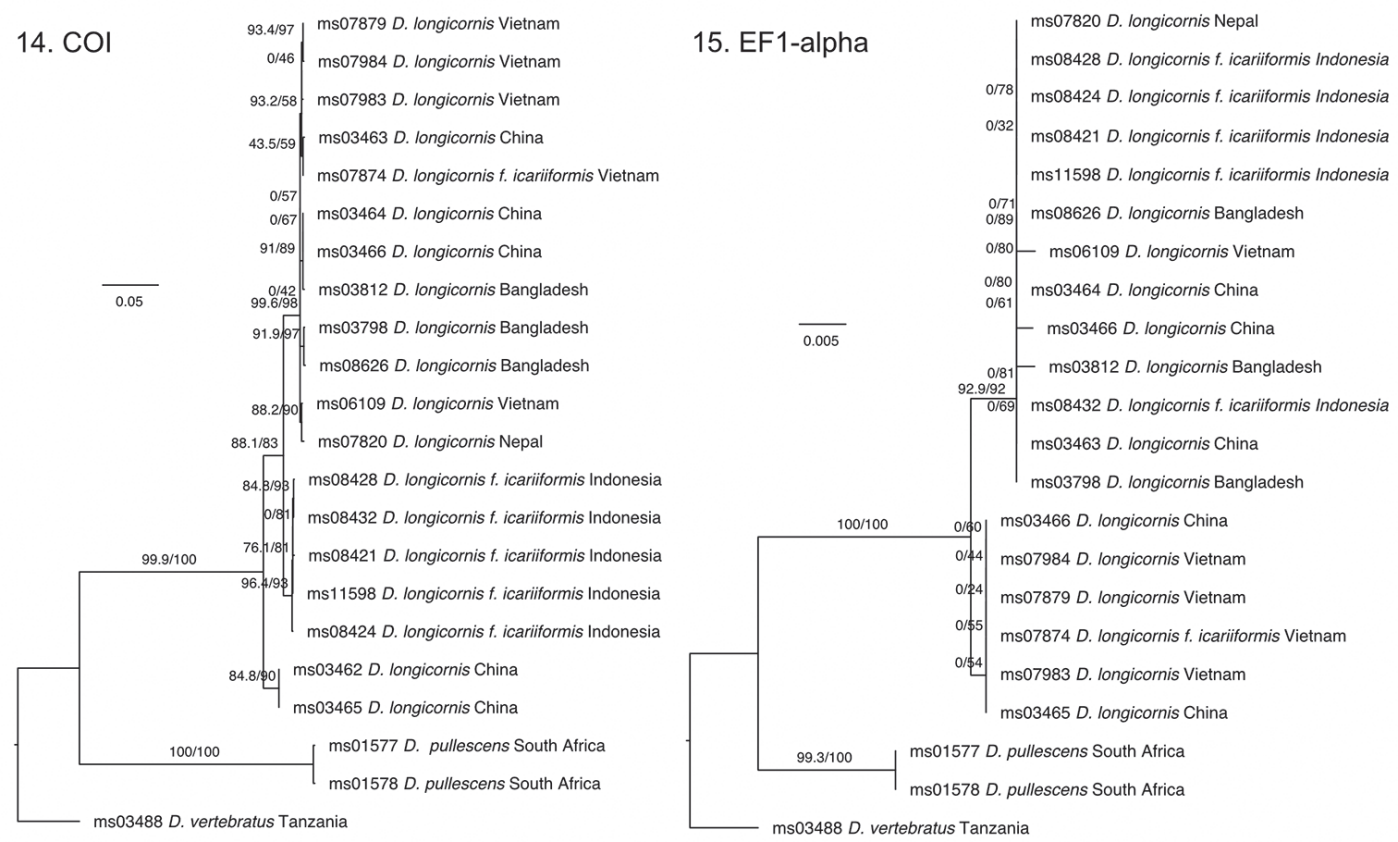
Maximum Likelihood trees based on COI (**14**) and EF1-alpha (**15**) DNA sequence data for *Dacus longicornis*, with *D. pullescens* Munro and *D. vertebratus* Bezzi as outgroups. Branch support values are rapid bootstrap values and approximate-likelihood ratio test values, scale bar indicates substitutions per site. Full details on the samples can be found in BOLD dataset DOI: http://dx.doi.org/10.5883/DS-DACSU.

*Dacus longicornis* Wiedemann is a widespread Southeast Asian species that is a minor pest: the larvae feed on *Luffa*, *Trichosanthes* and some other Cucurbitaceae (Allwood et al. 1999; Drew et al. 1998; Hardy and Adachi 1954). The morphology of *D. longicornis* is most extensively treated by Drew et al. (1998), where many synonyms were established and the variability of the species was first documented. In particular, this was the first, and only, publication that noted two forms: “There are two forms of *D. longicornis*, one with and one without a small medial postsutural vitta” (Drew 1998). However, this knowledge was not incorporated in subsequent publications, such as the Drew and Romig (2013) treatment of the Southeast Asian fauna, nor the accompanying Drew and Romig (2016) identification keys. We here provide the first figures of both forms (Figs 8–13). The postsutural medial vitta is absent in Bangladesh specimens (Figs 8, 9), but always present in Sulawesi specimens (Figs 10–13), although sometimes indistinct (Fig. 10). The dark markings on the anterior sides of the abdominal segments are more pronounced in Bangladesh specimens of *D. longicornis*, and Bangladesh specimens have a dark band across the occiput, connecting the compound eyes. The variable presence or absence of a medial vitta is not known for any other Dacinae species, but with all data considered, we see no reason at present to establish this form as a new species. Both COI and EF1-alpha sequence data reveal some genetic substructure in *D. longicornis*, but the structure differs between the two markers and does not match with the morphological forms (Figs 14, 15). Drew and Romig (2013) had studied the type material of *D. icariiformis* Enderlein, from India, and concluded that of the three type specimens –no holotype had been designated– the female was actually *D. longicornis* and only the two males are now regarded as lectotype and paralectotype. This confusion indicates that it is difficult to distinguish *D. longicornis* from *D. icariiformis*, and we here refer to the specimens of *D. longicornis* with a medial vitta that are genetically indistinguishable as D. longicornis form icariiformis. Because we did not study any type material, we refrain from synonymizing *D. icariiformis* with *D. longicornis*. However, we note that there are no diagnostic characters indicated in the literature to distinguish D. longicornis form icariiformis from *D. icariiformis*.

##### Sulawesi *Bactrocera melastomatos*

**Figures 16–19.**
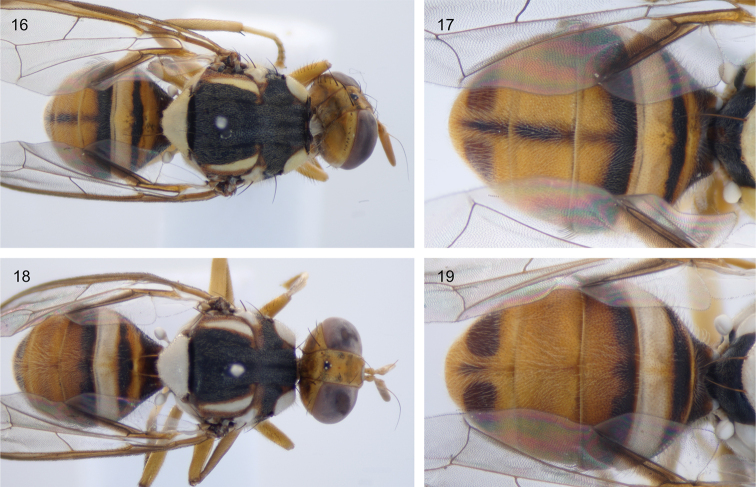
Sulawesi *Bactrocera melastomatos* resemble sympatric *Bactrocera usitata***16** specimen ms09144 *B. usitata*, dorsal view **17** close up of abdomen of ms09144 **18** specimen ms08838 *B. melastomatos*, dorsal view **19** close up of abdomen of ms08838.

**Figures 20, 21.**
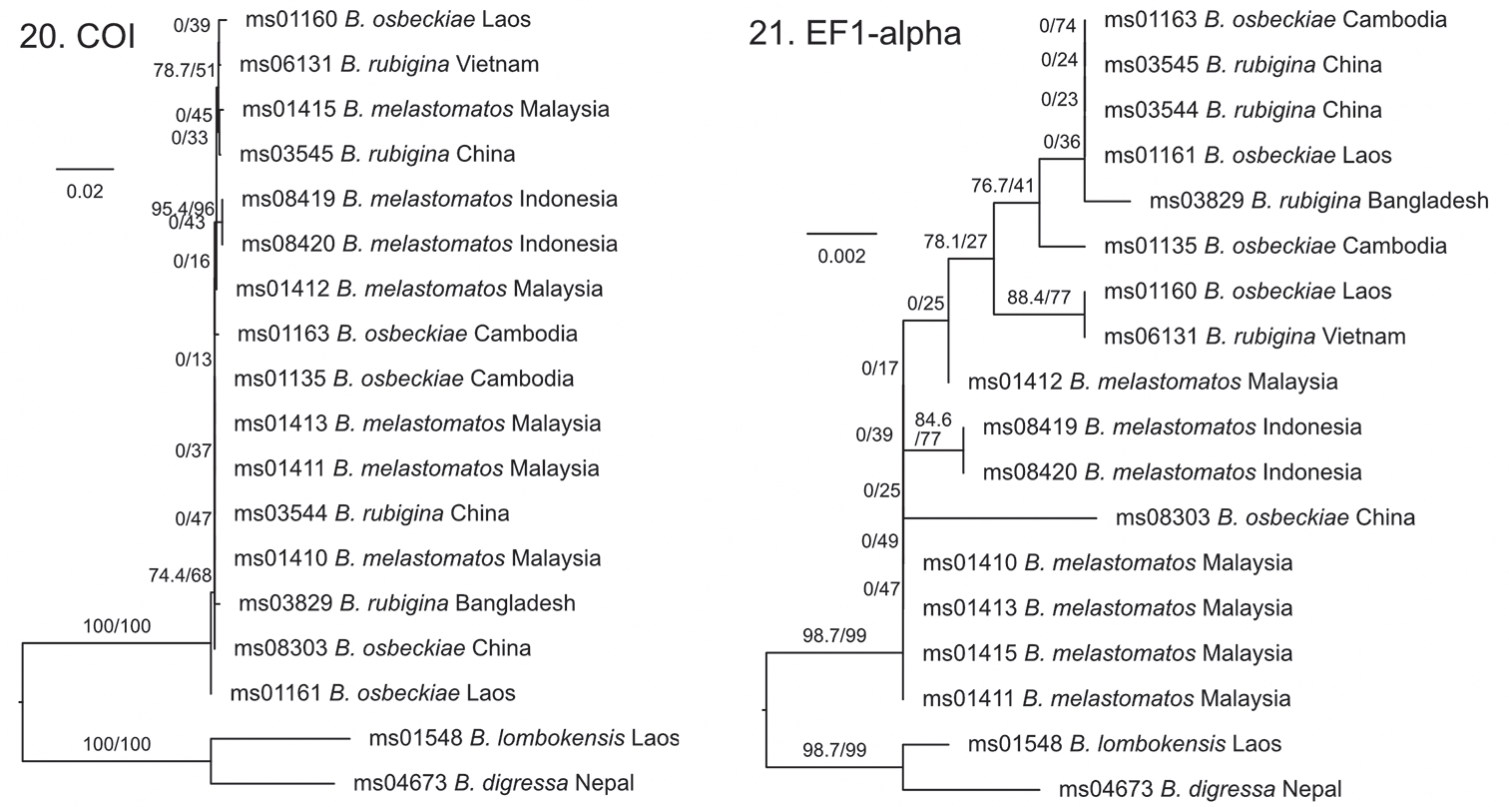
Maximum Likelihood trees based on COI (**20**) and EF1-alpha (**21**) DNA sequence data for *Bactrocera melastomatos* and allied species, using *B. lombokensis* Drew & Hancock and *B. digressa* Radhakrishnan as outgroup. Branch support values are rapid bootstrap values and approximate-likelihood ratio test values, scale bar indicates substitutions per site. Full details on the samples can be found in BOLD dataset DOI: http://dx.doi.org/10.5883/DS-DACSU.

We collected more than 300 specimens with a uniform morphotype that are tentatively included in the checklist as *B. melastomatos* (Table 1, Suppl. material 1: Table S1, Figs 16–19). We sequenced COI and EF1-alpha fragments for multiple specimens: they are genetically indistinguishable from specimens morphologically identified as *B. rubigina* (Wang & Zhao, 1989), *B. melastomatos* and *B. osbeckiae* Drew & Hancock, 1994 in both markers (Figs 20, 21). Morphologically, the specimens from Sulawesi are an imperfect fit for all three genetically suggested candidate species. Instead, they are more similar to the sympatric *B. usitata* Drew & Hancock, 1994 (Figs 16–19), but *B. usitata* has a medial black line across abdominal segments III–V, forming the typical *Bactrocera* black ‘T’, which is never present in Sulawesi *B. melastomatos*. The costal band of *B. usitata* and *B. melastomatos*, including the Sulawesi specimens, extends to vein R_4+5_, a character also shared with the southern Vietnam form of *B. rubigina* (Drew and Romig 2013). In the Drew & Romig (Drew and Romig 2016) identification keys, the absence of a black ‘T’ shape on the abdomen will lead to *Batrocera latifrons*, but that species is not attracted to cue lure, and has parallel yellow lateral postsutural vittae, which are strongly tapering, almost triangular, in Sulawesi *B. melastomatos*. Because there is no genetic support at present to describe this taxon as a separate species, we interpret the morphology of Sulawesi specimens as regional variation and leave their designation as *B. melastomatos* until more (genomic) data becomes available. Rearing specimens from host fruit would present important ecological data; the currently recorded hosts for this group are all Melastomataceae or Lauraceae (Liang et al. 1993; Allwood et al. 1999).

##### *Bactrocera carambolae* Drew & Hancock, 1994

**Figures 22–25.**
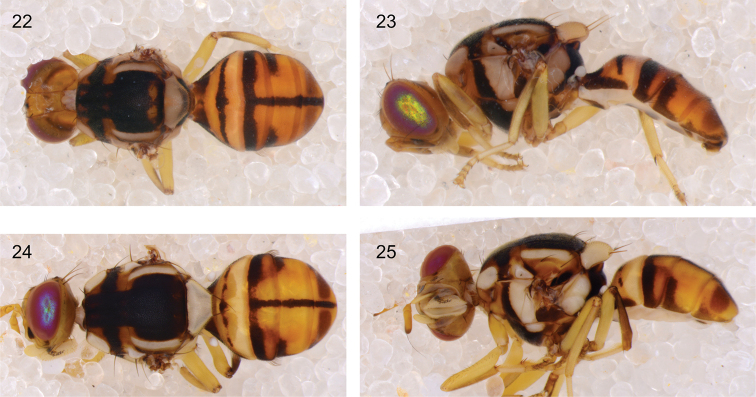
The two specimens of *Bactrocera carambolae* that represent the first records for Sulawesi, photographed in ethanol (wings were removed) **22** dorsal view of specimen ms08439 **23** lateral view of specimens ms08439 **24** dorsal view of specimen ms10710 **25** lateral view of specimen ms10710. Both specimens have the typical rectangular black mark on the lateral sides of the fourth abdominal segment, but lack the black mark on the fore femur, which can further help to distinguish *B. carambolae* from *B. dorsalis*.

We collected two specimens of *Bactrocera carambolae*, both at the Insitu locality, which represent the first records for Sulawesi (Figs 22–25). The morphology of the collected specimens is consistent with the description (Drew and Hancock 1994). However, because *B. carambolae* is morphologically very similar to *B. dorsalis* and they have intermingled mitochondrial DNA (San Jose et al. 2018b), we confirmed the identification with EF1-alpha DNA sequences. EF1-alpha is diagnostic for this species pair based on five positions (see also Leblanc et al. 2019). *Bactrocera carambolae* was already known from Java and Borneo (Vargas et al. 2015), so its presence in Sulawesi could be through natural dispersal, and it may have been missed during previous surveys. Alternatively, it could have been introduced through fruit transport, as it is a pest species on commercial fruit. Its natural distribution includes Vietnam, Laos, Cambodia, Thailand, Malaysia, and Indonesia (Java and Kalimantan), and it was recently reported in Bangladesh (Leblanc et al. 2019). In addition, it is highly invasive in agricultural areas in the Guianas in South America, where it is the only representative of Dacini.

##### *Dacus pullus* (Hardy 1982)

**Figures 26–31.**
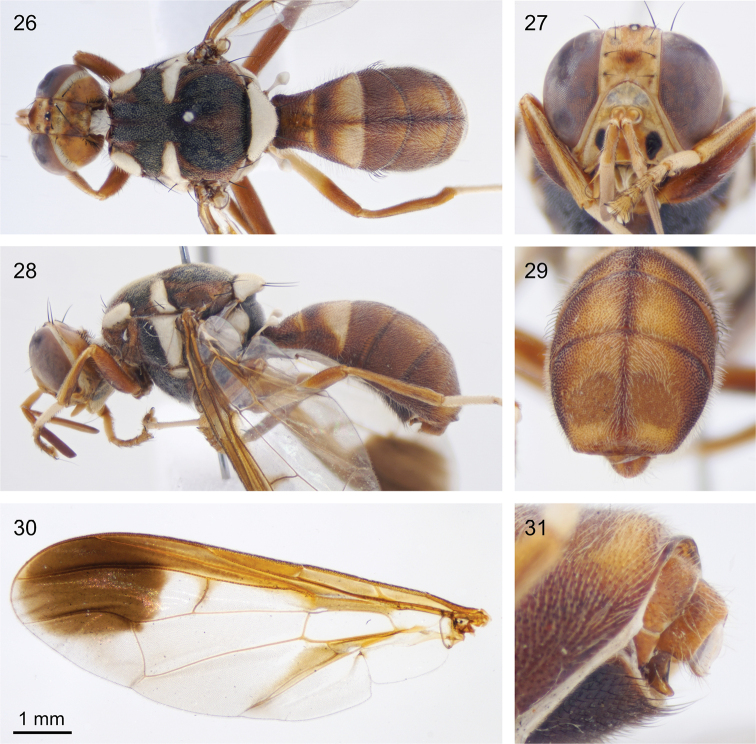
*Dacus pullus***26** dorsal view **27** frontal view of the face **28** lateral view **29** posterior view of the abdomen showing the ceromae **30** dissected wing **31** lateral close-up of the genitalia.

We record specimen ms09122 as a representative of *Dacus pullus* (Figs 26–31), although the wing markings are somewhat incongruent with the description and illustration of the only known other specimen of this species. The original species description states: “Costal band broad extending through upper half of cell Rs for its entire distance and expanded in apical portion to fill entire wing apex below upper edge of cell 2^nd^ M2” (Hardy 1982). However, the illustration does not depict an expansion in the apical portion. Drew and Romig (2013) also illustrated the holotype, again not showing a significant apical expansion of the costal band. The costal band of specimen ms09122 is mostly confined by vein R_4+5_, although there is infuscation of crossvein r-m, and distally expands to cross vein M. We opt to err on the side of caution and interpret this as intraspecific variation, and do not describe this specimen as a separate species, also considering the limited availability of material and the fact that both specimens were collected in Sulawesi.

## Discussion

### Between the lines

Sulawesi is a dispersal crossroads for the biotas of Southeast Asia, Australia, and Oceania. The updated species checklist we present here shows that Sulawesi is unique, with many endemic species, but that there are also strong connections with Southeast Asia, at least for the taxa under study. This finding does not support the earlier working hypotheses that posited a closer connection to the Sahul fauna, including Papua (Hardy 1982). Based on this recent data, it seems likely that Wallacea has been a “stepping-stone” for Dacini to reach Australasia and Oceania; 30 Sulawesi species are also found in (former) Sunda, while only one is shared with Sahul. This can provide crucial insight into the timing of the diversification of the group in the latter areas. Wallacea, and Sulawesi with it, was separated in discontinuous landmasses with fluctuating sea-levels until the mid-Miocene, 10–15 Ma (Hall 2009). Before this connection, Sunda and Sahul were separated by vast oceanic distances that were unlikely to be crossed by fruit flies. There are currently hundreds of Dacini species known from Australia and Oceania (Drew 1989, Drew and Romig 2001), which may have resulted from rapid radiation after reaching these new areas of ecological opportunity. Similarly, the timing of the formation of Wallacea suggests a relatively recent origin for the 34 Sulawesi endemic Dacini species. However, it should be noted that the Dacini fauna of Papua is understudied (White and Evenhuis 1999) and further surveys in this area may reveal shared geographic ranges with some of the species now presumed to be Wallacean endemics.

It has been advocated by some that the categorization of biogeographic regions should follow more quantitative measures (Kreft and Jetz 2010), as opposed to the qualitative assessments from the early explorers. Using a non-metric multidimensional scaling approach across a wide range of taxa, Kreft and Jetz (2010) suggested that Lydekker’s line was most appropriate for separating the Oriental region from Australasia. This agrees with our findings for Dacini, although, depending on the scale of the patterns in question, maintaining Wallacea as a separate biogeographic region can equally well be argued. Other authors have further included a phylogenetic component in the delimitation of biogeographic areas, which resulted in a suggested split of Wallacea: Sulawesi and the Lesser Sunda Islands were grouped with Southeast Asia into the Oriental region, whereas the Moluccas were grouped with Papua in the Oceanian region (Holt et al. 2013). However, none of these broad-scale assessments of biogeographic categorization include or consider invertebrate taxa. Moreover, as phytophagous insects, it might be expected that the biogeographic pattern of Dacini more closely tracks phytogeographic regions. A broad study that included 7,340 plant species across Southeast Asia suggested ‘central Wallacea’ [defined to encompass the Philippines, Sulawesi, lesser Sunda islands, Moluccas, and Java] as a separate region (van Welzen et al. 2011). This categorization is further corroborated by the climatic conditions; central Wallacea has a yearly dry season and monsoon, whereas both neighboring regions lack a prolonged dry season. For Dacini, we find few connections between the Philippines and Wallacea, but there are some, e.g., *B. commensurata*. Future surveys of the Papuan Dacini fauna, and placement of the Wallacean taxa in a phylogenetic framework, can further inform which biogeographic delimitation fits best with this group of fruit flies, and it is clear that more studies on invertebrate groups will be important to fully understand the biogeographic affinities of the islands that connect Asia with Australia and Oceania.

### Pests

As a tropical island, Sulawesi has a rich diversity of fruiting plants and, consequently, insects that utilize them. Our surveys were performed in cacao plantations; the only Dacini that is known to feed on cacao is the polyphagous *Bactrocera dorsalis* (Allwood et al. 1999). However, we have never observed flies attempting to oviposit on cacao nor have we found maggots inside the pods. Cacao is likely a very rare host for *B. dorsalis*, if at all, and potentially only fallen and dehiscent fruit where the tough skin is cracked is susceptible. We commonly found *B. dorsalis*, but also another pest species; *B. albistrigata* at all sites. Together they made up 70.6 % of all individuals collected. The host records of *B. albistrigata* include jackfruit (*Artocarpus heterophyllus*), jambu putik (*Syzygium* sp.), mango (*Mangifera indica* L.), guava (*Psidium guajava* L.) and rose apple (*Syzygium* sp.) some of which were planted near the cacao. It is interesting to note that although we encountered the cucurbit pest *Zeugodacus cucurbitae*, it was surprisingly rare. Possibly, this is due to the limited availability of melon hosts in the area (none were observed), although it is also known to feed on papaya, which were recurrent in the cacao orchards sampled, and non-commercial cucurbits that commonly occur as weeds in gardens and plantations. We further encountered small numbers of *B. umbrosa*, a pest of breadfruit (*Artocarpus altilis*) that has an extraordinarily wide distribution; it is the only species in the checklist that is known to be naturally dispersed across Southeast Asia, Wallacea, Australia and Oceania (Krosch et al. 2018). We suggest that, if desired, the population densities of pests in our survey areas can likely be decreased significantly with sanitation measures, most importantly the removal of fallen fruit and pruning of damaged fruit unfit for consumption.

## Supplementary Material

XML Treatment for Bactrocera (Bactrocera) niogreta

## Supplementary materials

Supplementary material 1 Table S1 Data type preadsheet with trapping records Explanation note Table including the lot numbers for each trapping event, fly identification, fly counts, lure used and exact locality. https://binary.pensoft.net/file/459314Camiel Doorenweerd, Arni Ekayanti, Daniel Rubinoff
